# Comparison of learning performance of 2 intubating laryngeal mask airways in novice: A randomized crossover manikin study

**DOI:** 10.1097/MD.0000000000006905

**Published:** 2017-05-12

**Authors:** Zi-Jia Liu, Jie Yi, Wei-Yun Chen, Xiu-Hua Zhang, Yu-Guang Huang

**Affiliations:** Department of Anesthesiology, Peking Union Medical College Hospital, Peking Union Medical College and Chinese Academy of Medicine Science, Beijing, China.

**Keywords:** laryngeal mask airway, manikin, novice

## Abstract

**Background:**

Intubating laryngeal mask airways (LMAs) such as i-gel and Aura-i could serve as rescue devices in resuscitation and further ensure the airway by facilitating trachea intubation without ventilation interruption. But data regarding intubating LMAs in novice are limited and skill degeneration without regular training has not been evaluated. So we designed this prospective randomized crossover manikin study to compare the learning performance of 2 intubating LMAs (i-gel and Aura-i).

**Methods:**

In total, 46 novice doctors participated in this study. After standardized training and finishing 3 consecutive successful intubations with both LMAs on manikin, each participant applied intubation with both LMAs in random order for initial evaluation. To evaluate skill retention, participants were reassessed 90 days later on the same manikin without retraining between times. Primary outcome was time to successful ventilation (TTV).

**Results:**

The TTV for i-gel was significantly shorter than Aura-i (initial evaluation 11.8 ± 2.9 seconds vs 22.4 ± 5.2 seconds, 90-days reevaluation 14.9 ± 3.6 seconds vs 28.9 ± 10.0 seconds, initial evaluation, *P* = .001; second evaluation, *P* < .001); during re-evaluation, TTV taken for i-gel and Aura-i were both significantly longer (initial evaluation, *P* = .001; second evaluation, *P* < .001) and ease score of insertion both increased profoundly (i-gel *P* = .025; Aura-i *P* < .001). In both assessments, participants preferred i-gel as easier alternative (initial evaluation, *P* = .001; second evaluation, *P* < .001). There was no difference in successful intubation rate, first attempt success rate, bronchoscopy assessment, and insertion score for 2 LMAs.

**Conclusion:**

Compared with Aura-i, i-gel showed a faster and easier intubation by novice doctors in this manikin study; the skill retention of intubation performance after 3 months was acceptable for both intubating LMAs, but TTV prolonged significantly.

## 1 Introduction

The incidence of cardiopulmonary resuscitation (CPR) has been reported as 1 to 4 cardiac arrests per 1000 patients admissions.^[1,2]^ However, in-hospital resuscitation is expected to be less efficient in general wards than in operation room and intensive care unit, partially because of insufficient trained staff.^[3,4]^ Airway patency during CRP is the key skill that needs to be improved in general wards.^[5]^

Tracheal intubation as current standard for airway management is a relatively difficult skill to acquire and a risky procedure when performed by nonanesthetic personnel.^[5,6]^ The AHA 2010 CRP guidelines suggest supraglottic airway device (SAD), especially the laryngeal mask airway (LMA), as valuable alternatives in airway management strategy during CPR.^[7,8]^ A number of reports suggest that LMA has valuable intubation performance in emergent airway management such as CPR, not only in the hands of those specialized healthcare providers, but also in those inexperienced with tracheal intubation.^[5,9–11]^

The i-gel (Intersurgical Ltd, Wokingham, Berks, UK) was a truly anatomical LMA without an inflatable cuff, while the Aura-i (Ambu Ltd, St Ives, Cambridge shire, UK) was a newly available LMA with integral features such as wide lumen and anatomical curvature to allow tracheal intubation. So intubating LMAs like i-gel and Aura-i were not only rescue devices during resuscitation, but also could further secure the airway on account of facilitating tracheal intubation without ventilation interruption.^[12,13]^

Even though the use of LMAs by novice doctors has been studied in several manikin studies,^[9,14–16]^ data regarding intubating LMA is limited and skill degeneration without regular training has not been evaluated yet. We therefore designed this study to compare the 2 intubating LMAs (i-gel and Aura-i) in the settings of randomized crossover manikin trial by novice doctors, to assess the learning performances, as well as to determine the ability retained to deploy the intubating LMAs after 3 months.

## 2 Methods

### 2.1 Institutional review board and informed consent

It was a randomized controlled crossover trial with written informed consent regarding the study purpose obtained from all participants. This study did not require approval of local Institute Ethics Committee, as a study of manikin with volunteers.

### 2.2 Enrollment of participants

In total, 46 nonanesthetic doctors of Peking Union Medical College Hospital participated in the study. The study was conducted between January 2016 and April 2016 and finally 44 doctors finished the assessment of skill retention 3 months later. To ensure that participants were true novice users of LMA, all of them had not previously used or had not been formally instructed how to use any type of SAD in experimental or clinical situations. And those who had experience of tracheal intubation were excluded.

### 2.3 Manikin and airway devices

The manikin used was as a standard airway adult trainer (Laerdal, Sentrum, Stavanger, Norway), placed in “sniffing position” (head tilted, chin lifted, and neck flexed).

The 2 airway devices used in this study were i-gel and Aura-i. Prior to beginning, an attending anesthesia consultant attempted all available adult-sized i-gel and Aura-i on the manikin, to define the correct size of devices. The size 4.0 i-gel and Aura-i were found to be most appropriate and selected. Furthermore, these sizes were in accordance with data from previous studies of the same manikin.^[11]^ The recommended complementary cuff volume of Aura-i to fit the manikin's larynx was 20 mL of air. The necessary equipment for each simulation was placed next to the manikin's head.

### 2.4 Study protocol

All participants were educated with a 5-minute video and a 15-minute presentation by the same attending anesthetist regarding the instructions and demonstrations for each LMA device, including manufacturer's manuals for each device. Afterward, all subjects were asked to practice on the manikin with each LMA device under guidance of the same senior anesthetist until 3 consecutive successful intubations with both devices were achieved. The participants were not allowed to watch others during insertion attempts, to avoid any learning effect throughout the whole process. Both devices and the manikin were well lubricated according to manufacturer's instructions.

Then each participant was asked to stand at the head of the manikin, inserted each of the 2 LMAs in a randomized sequence. Randomization was achieved using sealed numbered envelopes, generated 46 integers of 1 (i-gel first), or 2 (Aura-i first) by computer. Then, 2 different groups occurred to crossover.

**Figure 1:**
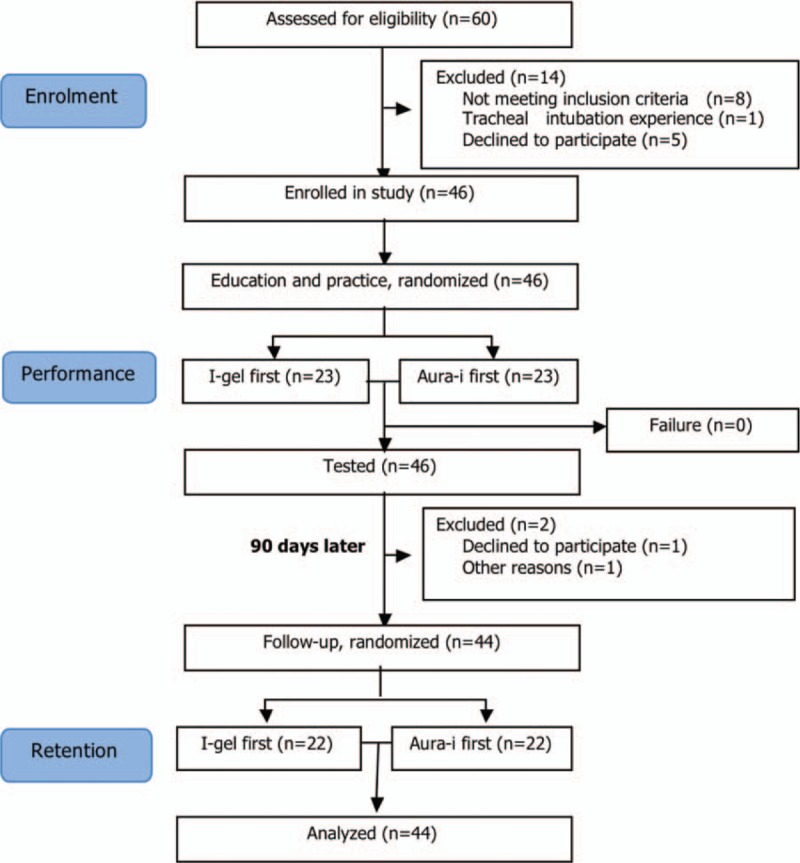
Study design flowchart.

Three months later, a second assessment was conducted to evaluate skill retention for those achieved successful ventilation with both LMAs, without additional demonstration or practice before it. Participants who performed LMA insertion during the 3-month period were excluded. The flowchart of study protocol was shown in Fig. 1.

### 2.5 Measurement definitions

The primary end point was duration of insertion, as to assess the immediate and long-term performance. That was time to successful ventilation (TTV), which was defined as the time from the moment that participant picked up a device to the initiation of ventilation, determined as visible expansion of manikin's lungs after connecting the LMA to a bag respirator.

The secondary end points were the successful intubation rate, first attempt success rate, gastric inflation, ease of insertion, bronchoscopy, and the insertion score. Removal of LMA from the mouth of manikin was considered an attempt. Failed insertion was defined as failure in inflating manikin's lungs within maximum of 3 attempts or a trial exceeding 60 seconds.

The LMA position was checked by a 3.8 mm fibrobrochoscope, which was introduced through the LMA and adjusted to obtain the best possible view of vocal cord. The view of vocal cord was scored as: grade 1, full view of vocal cord; grade 2, vocal cord partly visible; grade 3, only epiglottis visible; grade 4, no part of laryngeal structure visible.^[17]^

Calculation of insertion scores was achieved by assigning the score of 0 to 2 to each component of LMA insertion quality and security: fiber optic evaluation (0: grades 3–4, 1: grade 2, 2: grade 1); gastric insufflations of air (0: stomach expansion observed, 1: no stomach expansion); insertion times (0: thrice, 1: twice, 2: once). A summary of the scoring matrix is shown in Table 1. A single observer recorded all the time elapsed with the same stopwatch.

**Table 1:**
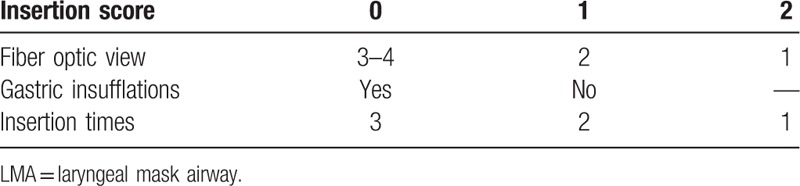
Insertion score of i-gel and Aura-i LMA (minimum score = 0, maximum score = 5).

Ease of insertion was a subjective evaluation graded with a numerical rating scale by participants to describe the difficulty their felt during the insertion. A 5-point rating scale was used: very easy to manipulate the device; easy to manipulate; neither easy nor difficult to manipulate; difficult insertion to manipulate; very difficult to manipulate the device.

### 2.6 Sample size and statistics analysis

Based on the results from the preliminary study, the mean insertion time (±standard deviation) required for i-gel and Aura-i was 15.2 ± 4.3 and 21.1 ± 7.6 seconds respectively. The difference time taken to insert i-gel and Aura-i was 5.9 seconds. Anticipated 6 seconds as the clinically meaningful difference between the 2 groups, 35 participants in each group were required for a crossover design to achieve a significance level of 0.05 and 90% power. Therefore, we recruited 46 patients in total to account for insertion failure in the first learning performance assessment and possible dropout.

Categorical data are presented as numbers (%), and continuous variables are expressed as mean ± standard deviation or the median and interquartile range, depending on type of distribution (data normality was tested with the K–S test). The difference in mean of TTVs was compared with Student *t* test. Success rate and percentage of gastric inflation were analyzed with *χ*^2^ test. The Mann–Whitney *U* test was applied to examine the bronchoscopy view, LMA insertion score, and ease of insertion variables. To evaluate the cumulative success rate associated with insertion time, Kaplan–Meier analyses were demonstrated. Statistical analysis was conducted using the SPSS 22.0 (SPSS Inc, Chicago, IL). The *P* < .05 (2-sided) was considered statistically significant.

## 3 Results

A total of 46 nonanesthetic novice doctors participated in the study, among whom 44 (31 females and 13 males, mean age 29 ± 5 years) finally attended both initial and skill retention assessments, including 22 internal physicians, 18 gynecologists, and 4 surgeons. Two subjects (0.4%) dropped out in follow-up.

### 3.1 Primary outcome

**Figure 2:**
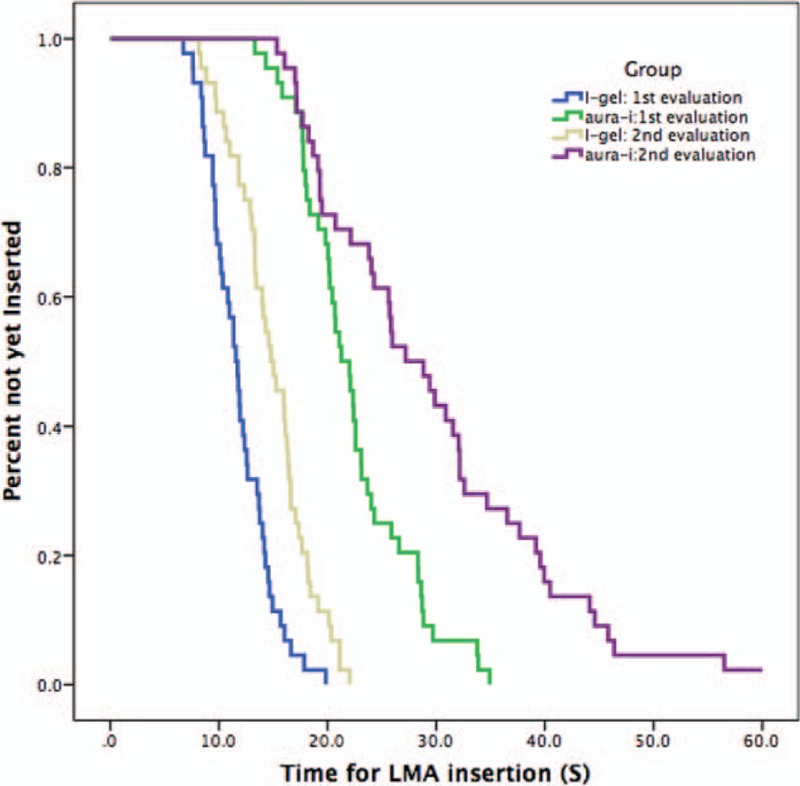
Kaplan–Meyer survival curve shows the percentage of participants still not having inserted the LMA successfully at various times. Forty-four doctors participated both in the initial and second evaluation. All of them inserted 2 devices successfully in the initial assessment. One participant failed to insert Aura-i LMA within 3 attempts in the skill retention assessment. LMA = laryngeal mask airway.

The TTV for i-gel was significantly shorter than Aura-i both at the first examination (11.8 ± 2.9 seconds vs 22.4 ± 5.2 seconds, *P* < .001) and the 90-day reassessment (14.9 ± 3.6 seconds vs 28.9 ± 10.0 seconds, *P* < .001; with 1 participant failed to insert Aura-i for the second evaluation). In follow-up, times taken for i-gel and Aura-i were both significantly longer than the initial training (for i-gel, 11.8 ± 2.9 seconds vs 14.9 ± 3.6 seconds, *P* < .001; for Aura-i, 22.4 ± 5.2 seconds vs 28.9 ± 10.0 seconds, *P* < .001). Cumulative success rates related to time to successful ventilation were illustrated by the Kaplan–Meyer survival curve (Fig. 2).

### 3.2 Secondary outcomes

#### 3.2.1 Total intubation success rate and first attempt success rate

For the immediate evaluation after training, no significant differences were shown in success rates and first attempt success rate between 2 LMAs. All participants achieved successful insertion with first attempt with Aura-i, while 98% participants inserted i-gel successfully in their first attempts (Table 2).

**Table 2:**
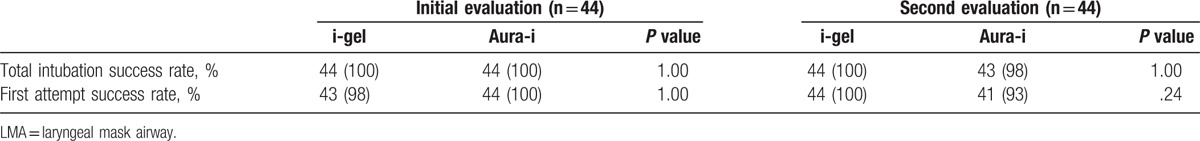
Success rate for insertion of i-gel and Aura-i LMA.

For the follow-up trail after 90 days, i-gel (100%) and Aura-i (98%) showed similarly high rate of total insertion success. Only 1 doctor failed to insert Aura-i after 3 attempts because of improper positioning. First-attempt insertion success rate of 2 devices was also comparable (Table 2).

#### 3.2.2 Gastric inflation, bronchoscopy assessment, and LMA insertion score

More gastric inflation during ventilation was seen in Aura-i group in both initial evaluation (*P* = .005) and reassessment after 90 days (*P* = .002) (Table 3).

**Table 3:**
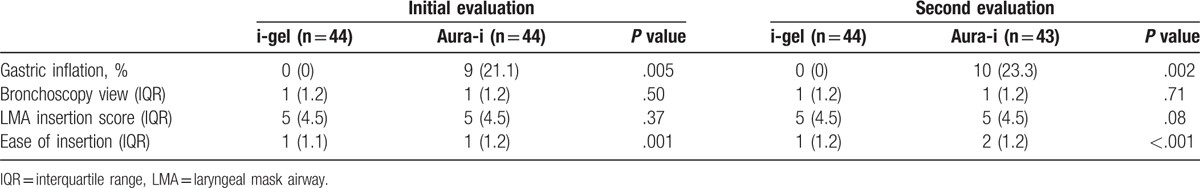
Bronchoscopy view, LMA insertion score, and ease of insertion of i-gel and Aura-i LMA.

No significant differences in the view of the glottis confirmed by bronchoscopy were found between 2 groups (i-gel: 66% grade 1, 32% grade 2, 2% grade 3; Aura-i: 73% grade 1, 25% grade 2, 2% grade 3, *P* = .50). In the 90-day reevaluation, satisfactory view of the glottis was also achieved with both the devices (i-gel: 64% grade 1, 36% grade 2; Aura-i: 67% grade 1, 33% grade 2, *P* = .71) (Table 3).

**Figure 3:**
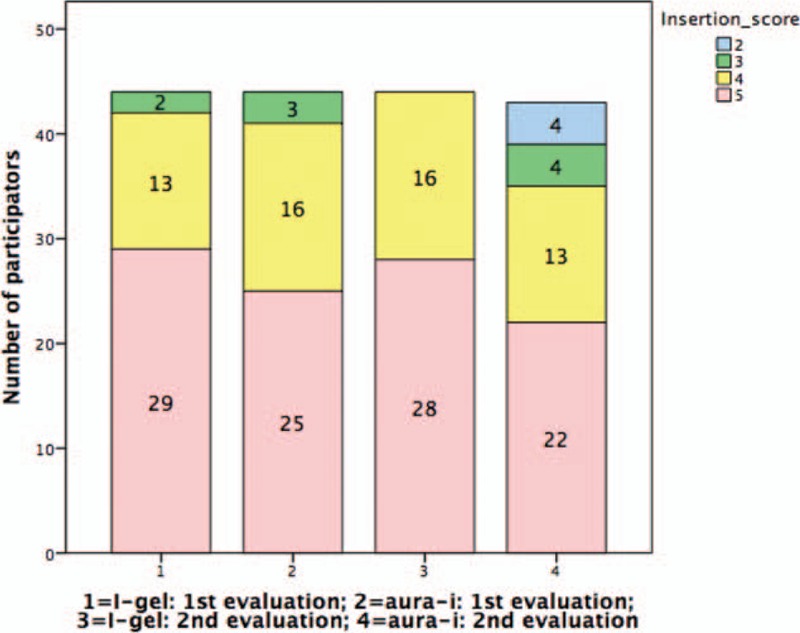
LMA insertion score of i-gel and Aura-i. Initial evaluation: *P* = .37; second evaluation: *P* = .08. LMA = laryngeal mask airway.

During the initial assessment, the participants performed comparably high LMA insertion score with both LMAs. Insertion score for i-gel during second evaluation seemed better compared with Aura-i, though no statistically significance was shown (*P* = .08) (Table 3, Fig. 3).

#### 3.2.3 Ease of insertion

**Figure 4:**
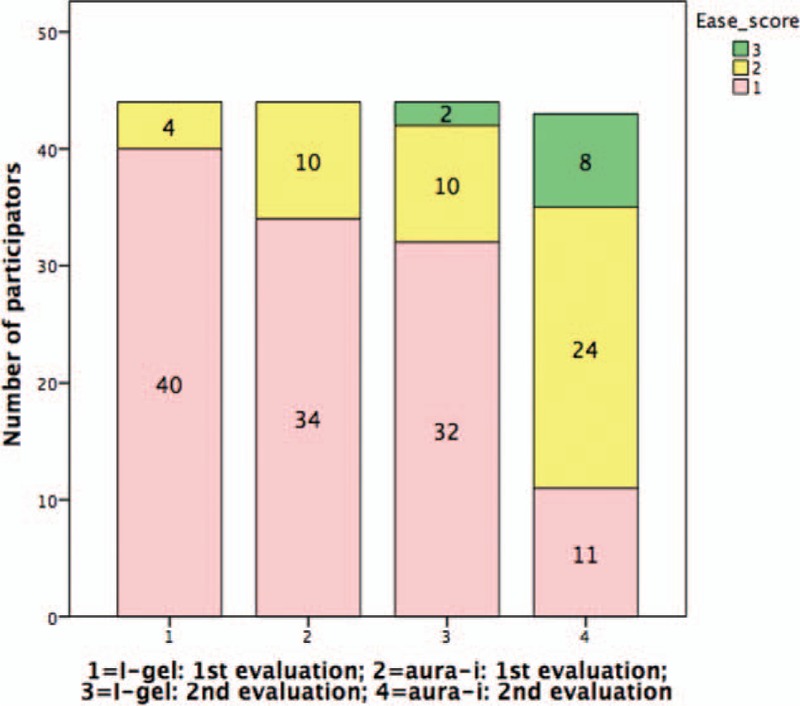
Ease of LMA insertion by participants was measured on a 5-point scale. The scale was marked from “very easiest” (1) to “very difficult” (5). i-gel: 1st versus 2nd evaluation *P* = .025; Aura-i: 1st versus 2nd evaluation *P* < .001; initial evaluation: i-gel versus Aura-i *P* = .001; second evaluation: i-gel versus Aura-i *P* < .001. Groups were compared using the Mann–Whitney test. LMA = laryngeal mask airway.

Regarding ease of insertion, participants reported that it was harder to insert the LMAs at reassessment (i-gel: *P* = .025; Aura-i: *P* < .001). Comparing the 2 LMAs, the participants reported i-gel was significantly easier to manipulate than Aura-i for both initial (*P* = .001) and second evaluations (*P* < .001) (Table 3, Fig. 4).

## 4 Discussion

Successful and effective airway management is of essential importance for CPR. Although tracheal intubation is the most widely used method for providing and maintaining secure airway, which is difficult to learn for those without airway experience and has unacceptably high failure rate causing substantial morbidity and mortality in novice hands.^[18,19]^ bag mask ventilation (BMV) is associated with high possibility of regurgitation and aspiration.^[20]^ In addition, both tracheal intubation and BMV have markedly decreased performance at follow-up in inexperienced hands without constant practice.^[21]^ However, LMAs may benefit when establishing airway in emergency situations by novice physicians. It has been proven that LMAs are easy and safe to use after short training period.^[15,16]^

However, the risk of regurgitation or aspiration will increase if the time to use LMAs prolonged, especially for patients with abnormal gastric empty or high airway pressure.^[22]^ It would be a wise choice to replace LMAs with endotracheal tube earlier, when prolonged mechanical ventilation needed. So we considered selecting intubating LMAs, which could serve as conduit for intubation. It will be safer and more convenient to conduct endotracheal intubation via LMAs inner lumen without interrupting ventilation or compression in CPR, particularly for difficulty airways.^[23,24]^ Up until now limited data were available for which intubating LMA had better learning performance than others, especially in hands of novice. Therefore we designed this study.

The results from this study concealed with previous studies that LMAs have short insertion time for novice physicians.^[14]^ Stroumpoulis et al^[15]^ described the time of intubation for i-gel was 15.2 seconds for novice doctors. In a skill retention study for 3-year medical students, the application time of i-gel was 10 seconds during first attempt, and 12 seconds at follow-up assessment.^[21]^ LMAs stand substantially indirect way of airways establishment without visualization of vocal cords and surrounding structures.^[16]^ However, there were no studies reporting the insertion time to achieve lung ventilation for Aura-i in novice.

In our study, TTV with the i-gel was half of that with the Aura-i in both initial and reevaluation after 90 days. The i-gel was applied in less than 30 seconds in both assessments for all participants. The main reason might be that i-gel has a noninflatable cuff, so no further measures have to be taken after insertion. It has not been indicated how much time saving is “clinic significant” for airway establishment in emergency, especially in face of current guidelines emphasizing continuous chest compression.^[7,8]^ But shorter time to establish airways will directly shorten the time to deliver rescue breath, as well as detract less from more critical procedures as compression and defibrillation. So we suggest the i-gel might be more suitable among LMAs for emergency airway management.

It is of importance to remember that LMAs increase risks of aspiration compared with endotracheal intubation.^[25]^ So we considered gastric inflation in our study and found that it happened more frequently for Aura-i than predicted. The main reason was that participants often neglected to inflate the cuff and sometimes air inflated was insufficient. Although we advised 20 mL as ideal volume, difficulties might be met in practice. Sometimes, they ignored that the injector was not pushed into the cuff tightly. The i-gel can seal the laryngopharyngeal space without a cuff, which could reduce errors in handling. Nevertheless, the risk of regurgitation and aspiration remains and more clinical evidence should be gathered before LMAs can be recommended as a safe and effective primary alternative airway device in emergency airway situations such as CPR.

The high success rate in our study (100% within 2 attempts in initial examination) was consistent with other evidence related to i-gel and Aura-i when performed by novice operators.^[11,26]^ Both LMAs had high first-pass success rates, satisficing bronchoscopy view and good LMAs insertion score with little differences in this study. In the initial assessment, 98% novice doctors could complete successful ventilation with both LMAs during first attempt, acquiring bronchoscopy view grade 1 or 2. So we suggest that intubating LMAs could be introduced to general wards with high feasibility, even for physicians not proficient in airway management. They could achieve rapid insertion and effective ventilation with high success rate after short training session. Moreover, the vocal cords were almost fully visible through the LMAs by bronchoscope, which will benefit skilled physicians performing endotracheal intubation later through the LMAs conduit.

To our surprise, despite intubation performance comparable, there was significant difference in subjective rating of ease-to-use. Participants announced that i-gel was much easier to manipulate than Aura-i during both assessments. The reason for ease assessment was partly speed of insertion. The participants said: “We don’t need to inflate the balloon, so i-gel is much faster and easier to use,” “It's more intuitive to place i-gel, because you just pick it up and put into mouth.”

Airway management is likely to be infrequent for doctors in general wards, so skill acquisition and retention are both important for safe practice. At follow-up, i-gel and Aura-i showed comparable performance levels with the initial results in success rate, first-pass rate, bronchoscopy view, and LMA insertion score. Both LMAs were easier to handle and required less practice for their simple design. But TTV had markedly increased at the second assessment (i-gel increased by 26% and Aura-i 29%). In addition, the novice agreed that the initial intubation was easier both for i-gel or Aura-i, even though similar intubation performance was shown. One possible reason was that the novice doctors were not allowed to make any practice before the reevaluation. Consequently, we suggest that regular learning sessions and retraining may be helpful to improve self-confidence and skill, and further speed the insertion, although the skill retention was acceptable for both devices after 3 months.

This study had several limitations. First, we did not investigate the effectiveness of the 2 intubating LMAs as conduits for tracheal intubation. It is appropriate to perform endotracheal intubation through intubating LMAs utilizing fibrobrochoscope after recovery of spontaneous circulation.^[27]^ de Lloyd et al^[28]^ compared Aura-i and i-gel for fibreoptic-guided tracheal intubation in a manikin and showed quicker endotracheal intubation through i-gel and more failures through the Aura-i. Second, it was a manikin-based study that may not be definitively extrapolated to clinical application. The airway scenarios did not accurately simulate real intubation situations in resuscitation, such as blood, secretion, vomit, or trismus. Although the success rates, real time spent, and quality of ventilation may generally not be the same in actual patients,^[29]^ the differences between 2 intubating LMAs are likely to be analogous. Taking account of statistical design, the same manikin acted as a constant could allow strict standardization of placement for each LMA and each participant. So our results constitute a potential evidence for the use of intubating LMA for securing emergency airway in novice doctors. Additionally, the 2 missing participants in follow-up assessment might lead to potential bias.

## 5 Conclusion

We conclude that, in the hands of novice practitioners, both intubating LMAs evaluated could be placed accurately and successfully into manikin's airway after a brief training. The i-gel results in shorter insertion time and lower rate of gastric inflation compared with the Aura-i, and it is preferred as an easier device by participants. And the skill retention is acceptable 3 months later without retraining, except for the prolonged time to ventilation. We suggest i-gel may be a more efficient intubating LMA for novice physicians in managing airway and regular practice would benefit to maintain the skill.

## Acknowledgments

The authors acknowledge all the participants in this study to make the project possible. The authors gratefully thank Han Wei PhD for his support and help in statistical analysis. Without the organization work of Shengyu Zhang MD, they would not be able to execute the follow-up favorably.
